# Supplementary material for: Klebsiella pneumoniae Lipopolysaccharides Serotype O2afg Induce Poor Inflammatory Immune Responses Ex Vivo
Source: Microorganisms. 2021 Jun 17;9(6):1317. doi: 10.3390/microorganisms9061317 (PMC8234205; doi:10.3390/microorganisms9061317)
Supplement: Supplementary file 1 [file microorganisms-09-01317-s001.zip › microorganisms-1228089-supplementary.pdf]

microorganisms

MDPI

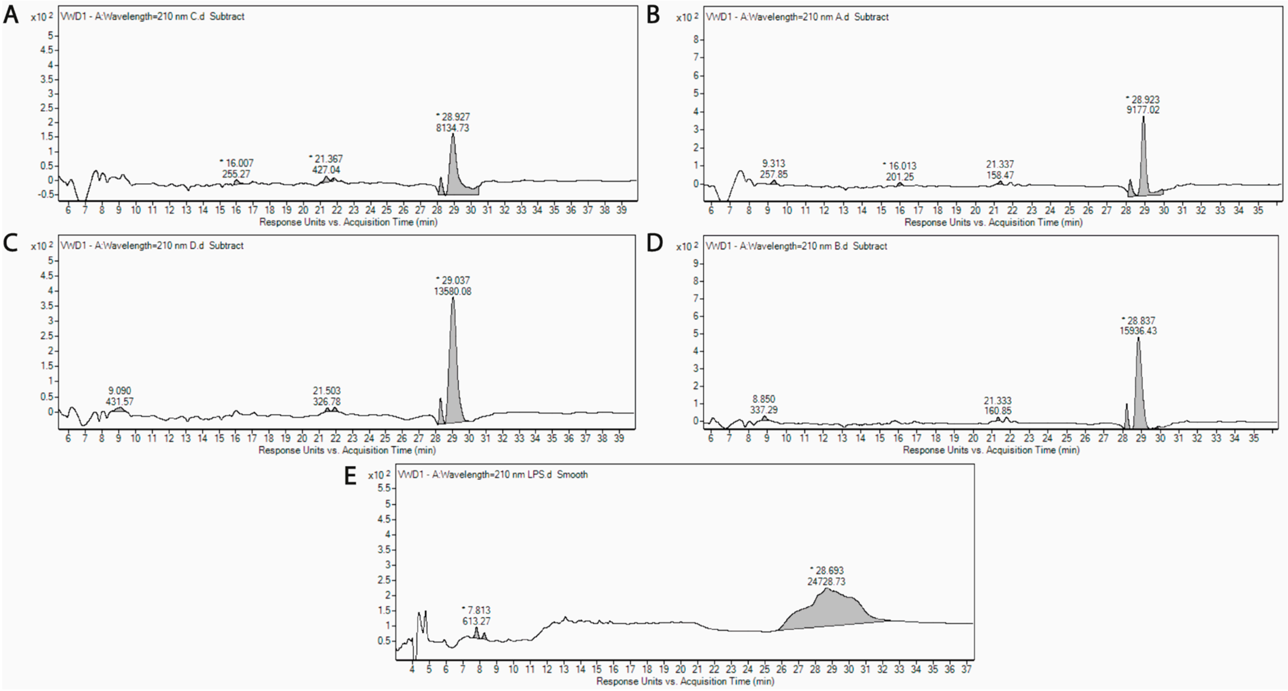

Figure S1. The purity of the purified LPS was determined by HPLC, based on the integrated areas of the peaks' ratio (on gray) corresponding to LPS in the chromatograms of (A) *K. pneumoniae* B5055 LPS O1, (B) *K. pneumoniae* C5046 LPS O2a, (C) *K. pneumoniae* 6613 LPS O2afg and (D) *K. pneumoniae* ST258 LPS O2afg. Commercial *K. pneumoniae* LPS O1 (E) was used as control.

Method. Briefly, samples were injected (injection volume = 10 µl) into a HPLC-UV/Vis system (Agilent 1260 Infinity; Agilent, Santa Clara, CA, USA) equipped with a reversed-phase C18 column (Phenomenex Luna, 150 mm x 4.6 mm, particle size 3 µm; Phenomenex, Torrance, CA, USA) outfitted with a C18 security-guard column (Phenomenex, 4mm x 3mm). The eluent phase was composed of analytical grade formic acid and HPLC/MS grade water 0.1% v/v (phase A), and analytical grade formic acid - HPLC/MS acetonitrile 0.1% v/v (phase B). Both column and security-guard column were maintained at 30 °C and the flow-rate was set to 0.5 mL/min using the following gradient: 0 - 5 min, 5% phase B isocratic; 5 - 15 min, linear gradient from 5% to 15% phase B; 15 - 20 min, 15% phase B isocratic; 20 - 25 min, linear gradient from 15% to 30% phase B; 25 - 35min, 30% phase B isocratic; 35 - 45 min, washing and reconditioning of the column to 5% phase B. The eluate was monitored measuring the absorbance at 210 nm.

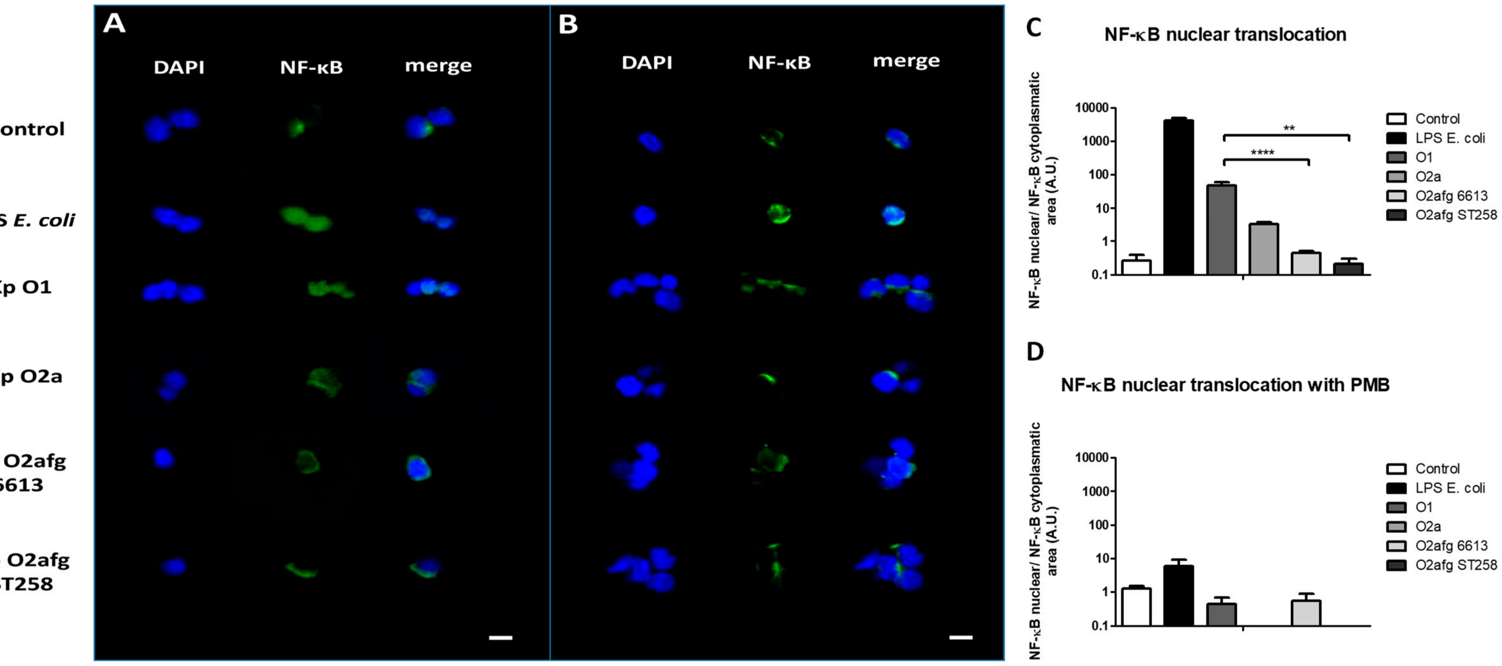

Figure S2. Translocation of NF-κB to monocytes' nuclei. (A and C) Stimulation of monocytes for 6 h with 1 μg/ml of each of the *E. coli* LPS and *K. pneumoniae* O1 and O2a LPS induces translocation of NF-κB to the nuclei. Conversely, in monocytes stimulated likewise with *K. pneumoniae* O2afg LPS, nuclear translocation of NF-κB is severely impaired. (B and D) Inhibition of NF-κB nuclear translocation with PMB. Incubation with PMB reduced almost completely NF-κB translocation in cells treated with *E. coli* LPS. Moreover, PMB fully reverted NF-κB nuclear translocation induced by *K. pneumoniae* O1 and O2a-antigen. Results are representative of approximately 10 fields and 40 cells per condition tested. Blue, DAPI; Green, NF-κB. Bar, 10 μm. Statistical analysis was done using the paired Student t-test, ** $p < 0,01$ and **** $p < 0,0001$.
