# Supplementary material for: Klebsiella pneumoniae Lipopolysaccharides Serotype O2afg Induce Poor Inflammatory Immune Responses Ex Vivo
Source: Microorganisms. 2021 Jun 17;9(6):1317. doi: 10.3390/microorganisms9061317 (PMC8234205; doi:10.3390/microorganisms9061317)
Supplement: Supplementary file 1 [file microorganisms-09-01317-s001.zip › Supplementary File 1 30 multiplex.pdf]

Table S1

Mono 48hr	EGF	SEM	Eotaxin	SEM	FGF-2	SEM	G-CSF	SEM	GM-CSF	SEM	HGF	SEM
NS	<=0	<=0	<=0	<=0	<=0	<=0	<=0	<=0	<=0	<=0	<=0	<=0
KPO1	<=0	<=0	<=0	<=0	<=0	<=0	<=0	<=0	<=0	<=0	<=0	<=0
KPO2	<=0	<=0	<=0	<=0	<=0	<=0	<=0	<=0	<=0	<=0	<=0	<=0
ST258	<=0	<=0	<=0	<=0	<=0	<=0	<=0	<=0	<=0	<=0	<=0	<=0
6613	<=0	<=0	<=0	<=0	<=0	<=0	<=0	<=0	<=0	<=0	<=0	<=0

Table S2

Mono 6hr	EGF	SEM	Eotaxin	SEM	FGF-2	SEM	G-CSF	SEM	GM-CSF	SEM	HGF	SEM
NS	0.33	0.2	<=0	<=0	<=0	<=0	3.76	0.17	0.28	0.1	<=0	<=0
NS + PMB	0.33	0.2	0.54	0.13	<=0	<=0	3.76	0.19	0.35	0.12	<=0	<=0
LPS	0.47	0.18	<=0	<=0	<=0	<=0	3.76	0.17	1.55	0.88	<=0	<=0
KPO1	0.61	0.21	<=0	<=0	<=0	<=0	3.76	0.15	0.36	0.12	<=0	<=0
KPO2	0.06	0.08	<=0	<=0	<=0	<=0	3.76	0.17	0.47	0.22	<=0	<=0
ST258	<=0	<=0	<=0	<=0	<=0	<=0	6.35	2.1	0.77	0.12	<=0	<=0
6613	<=0	<=0	<=0	<=0	<=0	<=0	1.38	0.5	0.7	0.33	<=0	<=0
LPS + PMB	0.33	0.16	1.45	0.77	<=0	<=0	4.37	0.2	0.32	0.12	<=0	<=0
KPO1 + PMB	0.75	0.11	0.95	0.64	<=0	<=0	5.05	2.1	0.33	0.12	<=0	<=0
KPO2 + PMB	0.2	0.01	0.33	0.1	<=0	<=0	1.38	0.5	0.32	0.12	<=0	<=0
ST258 + PMB	<=0	<=0	<=0	<=0	<=0	<=0	1.38	0.5	0.32	0.12	<=0	<=0
6613 + PMB	0.61	0.23	<=0	<=0	<=0	<=0	2.67	0.5	0.39	0.12	<=0	<=0

Table S3

WB 6hr	EGF	SEM	Eotaxin	SEM	FGF-2	SEM	G-CSF	SEM	GM-CSF	SEM	HGF	SEM
NS	1.19	0.6	0.33	0.31	<=0	<=0	1.38	0.55	0.32	0.12	<=0	<=0
NS + PMB	1.62	0.34	<=0	<=0	<=0	<=0	1.48	0.32	0.12	0.32	<=0	<=0
LPS	0.98	0.5	1.35	0.37	<=0	<=0	3.96	0.43	0.35	0.33	<=0	<=0
KPO1	1.5	0.2	0.95	0.23	<=0	<=0	3.36	0.67	0.34	0.16	<=0	<=0
KPO2	2.35	2.1	<=0	<=0	<=0	<=0	<=0	<=0	0.3	0.01	<=0	<=0
ST258	2.36	0.5	<=0	<=0	<=0	<=0	2.67	0.4	0.28	0.39	<=0	<=0
6613	3.11	0.5	<=0	<=0	<=0	<=0	1.38	0.45	0.42	0.1	<=0	<=0
LPS + PMB	1.03	0.45	<=0	<=0	<=0	<=0	1.16	1.2	0.32	0.32	<=0	<=0
KPO1 + PMB	2.84	0.17	<=0	<=0	<=0	<=0	3.06	2.1	0.34	0.26	<=0	<=0
KPO2 + PMB	3.93	0.18	<=0	<=0	<=0	<=0	3.76	1.28	0.28	0.11	<=0	<=0
ST258 + PMB	2.57	0.37	<=0	<=0	<=0	<=0	3.95	2.3	0.39	0.21	<=0	<=0
6613 + PMB	2.67	2.1	<=0	<=0	<=0	<=0	4.05	1.77	0.35	0.15	<=0	<=0

IFN alpha	SEM	IFN gamma	SEM	IL-1 beta	SEM	IL-10	SEM	IL-12p40	SEM	IL-13	SEM
<=0	<=0	<=0	<=0	<=0	<=0	<=0	<=0	<=0	<=0	<=0	<=0
<=0	<=0	<=0	<=0	30.59	1.33	44.14	<=0	34.19	0.27	<=0	<=0
<=0	<=0	<=0	<=0	<=0	<=0	<=0	<=0	<=0	<=0	<=0	<=0
<=0	<=0	<=0	<=0	<=0	<=0	<=0	<=0	<=0	<=0	<=0	<=0
<=0	<=0	<=0	<=0	<=0	<=0	<=0	<=0	25.21	0.33	<=0	<=0

IFN alpha	SEM	IFN gamma	SEM	IL-1 beta	SEM	IL-10	SEM	IL-12p40	SEM	IL-13	SEM
<=0	<=0	<=0	<=0	<=0	<=0	<=0	<=0	1.37	0.22	0.26	0.11
<=0	<=0	0.01	<=0	<=0	<=0	<=0	<=0	0.65	0.45	0.26	0.11
<=0	<=0	<=0	<=0	<=0	<=0	<=0	<=0	5.55	0.66	<=0	<=0
<=0	<=0	<=0	<=0	<=0	<=0	<=0	<=0	1.83	0.67	<=0	<=0
<=0	<=0	<=0	<=0	<=0	<=0	<=0	<=0	1.83	0.77	<=0	<=0
<=0	<=0	<=0	<=0	<=0	<=0	<=0	<=0	2.29	0.11	<=0	<=0
<=0	<=0	<=0	<=0	<=0	<=0	<=0	<=0	1.37	0.45	<=0	<=0
<=0	<=0	<=0	<=0	<=0	<=0	<=0	<=0	1.02	0.64	<=0	<=0
<=0	<=0	<=0	<=0	<=0	<=0	<=0	<=0	1.37	0.16	<=0	<=0
<=0	<=0	<=0	<=0	<=0	<=0	<=0	<=0	<=0	<=0	<=0	<=0
<=0	<=0	<=0	<=0	<=0	<=0	<=0	<=0	0.66	0.21	<=0	<=0
<=0	<=0	<=0	<=0	<=0	<=0	<=0	<=0	1.02	0.43	<=0	<=0

IFN alpha	SEM	IFN gamma	SEM	IL-1 beta	SEM	IL-10	SEM	IL-12p40	SEM	IL-13	SEM
0.4	0.33	<=0	<=0	<=0	<=0	<=0	<=0	2.64	0.175	<=0	<=0
1.79	0.12	0.01	<=0	<=0	<=0	1.12	0.6	<=0	0	<=0	<=0
2.23	0.37	<=0	<=0	28.58	1.18	0.02	<=0	58.8	2.25	<=0	<=0
3.17	0.23	<=0	<=0	10.41	0.515	2.38	0.7	40.8	0.8	<=0	<=0
0.83	0.32	<=0	<=0	29.73	0.37	0.55	0.22	10.76	0.495	<=0	<=0
0.4	0.36	<=0	<=0	22.55	0.535	<=0	<=0	4.08	0.14	<=0	<=0
0.83	0.47	<=0	<=0	31.96	1.56	<=0	<=0	8.2	0.35	<=0	<=0
0.83	0.28	<=0	<=0	18.33	0.63	1.12	0.31	14.96	1.83	<=0	<=0
0.83	0.77	<=0	<=0	8.265	0.175	<=0	<=0	4.72	0.483	<=0	<=0
0.4	0.32	<=0	<=0	4.99	0.21	<=0	<=0	2.64	0.315	<=0	<=0
2.66	0.31	<=0	<=0	32.53	1.23	<=0	<=0	18.68	1.09	<=0	<=0
0.83	0.34	<=0	<=0	38.04	1.24	<=0	<=0	22.2	0.45	<=0	<=0

IL-15	SEM	IL-17A	SEM	IL-1RA	SEM	IL-2	SEM	IL-2R	SEM	IL-4	SEM	IL-5
18.92	1.03	<=0	<=0	<=0	<=0	<=0	<=0	<=0	<=0	<=0	<=0	<=0
<=0	<=0	<=0	<=0	<=0	<=0	<=0	<=0	<=0	<=0	<=0	<=0	<=0
<=0	<=0	<=0	<=0	<=0	<=0	<=0	<=0	<=0	<=0	<=0	<=0	<=0
<=0	<=0	<=0	<=0	<=0	<=0	<=0	<=0	<=0	<=0	<=0	<=0	<=0
<=0	<=0	<=0	<=0	<=0	<=0	<=0	<=0	<=0	<=0	<=0	<=0	<=0

IL-15	SEM	IL-17A	SEM	IL-1RA	SEM	IL-2	SEM	IL-2R	SEM	IL-4	SEM	IL-5
4.18	0.55	<=0	<=0	<=0	<=0	<=0	<=0	<=0	<=0	<=0	<=0	<=0
1.66	0.32	<=0	<=0	<=0	<=0	<=0	<=0	<=0	<=0	<=0	<=0	<=0
6.9	0.43	<=0	<=0	<=0	<=0	<=0	<=0	1.64	<=0	<=0	<=0	<=0
5.73	0.67	<=0	<=0	<=0	<=0	<=0	<=0	<=0	<=0	<=0	<=0	<=0
6.73	0.45	<=0	<=0	<=0	<=0	<=0	<=0	0.72	<=0	<=0	<=0	<=0
5.67	0.33	<=0	<=0	0.3	<=0	<=0	<=0	<=0	<=0	<=0	<=0	<=0
3.17	0.12	<=0	<=0	<=0	<=0	<=0	<=0	<=0	<=0	<=0	<=0	<=0
3.4	1.2	<=0	<=0	<=0	<=0	<=0	<=0	<=0	<=0	<=0	<=0	<=0
6.09	2.1	<=0	<=0	<=0	<=0	<=0	<=0	<=0	<=0	<=0	<=0	<=0
4.47	1.28	<=0	<=0	<=0	<=0	<=0	<=0	<=0	<=0	<=0	<=0	<=0
4.34	2.3	<=0	<=0	<=0	<=0	<=0	<=0	<=0	<=0	<=0	<=0	<=0
4.01	1.77	<=0	<=0	<=0	<=0	<=0	<=0	<=0	<=0	<=0	<=0	<=0

IL-15	SEM	IL-17A	SEM	IL-1RA	SEM	IL-2	SEM	IL-2R	SEM	IL-4	SEM	IL-5
1.17	1.02	<=0	<=0	<=0	<=0	<=0	<=0	<=0	<=0	<=0	<=0	<=0
1.17	2.05	<=0	<=0	<=0	<=0	<=0	<=0	<=0	<=0	<=0	<=0	<=0
2.79	1.12	<=0	<=0	10.4	2.64	<=0	<=0	2.11	2.11	<=0	<=0	<=0
5.32	3.74	<=0	<=0	<=0	<=0	<=0	<=0	6.35	1.33	<=0	<=0	<=0
2.54	1.18	<=0	<=0	<=0	<=0	<=0	<=0	1.43	0.98	<=0	<=0	<=0
3.95	0.66	<=0	<=0	5.32	1.22	<=0	<=0	0.72	0.55	<=0	<=0	<=0
3.57	1.31	<=0	<=0	<=0	<=0	<=0	<=0	2.11	1.22	<=0	<=0	<=0
4.75	2.54	<=0	<=0	<=0	<=0	<=0	<=0	0.72	0.43	<=0	<=0	<=0
2.84	0.91	<=0	<=0	<=0	<=0	<=0	<=0	<=0	<=0	<=0	<=0	<=0
1.99	1.95	<=0	<=0	<=0	<=0	<=0	<=0	<=0	<=0	<=0	<=0	<=0
13.89	1.53	<=0	<=0	2.98	1.02	<=0	<=0	10.22	2.88	<=0	<=0	<=0
5.27	0.99	<=0	<=0	5.74	0.45	<=0	<=0	4.02	1.21	<=0	<=0	<=0

SEM	IL-6	SEM	IL-7	SEM	IL-8	SEM	IP-10	SEM	MCP-1	SEM	MIG	SEM
<=0	16.07	<=0	<=0	<=0	21063.49	<=0	<=0	<=0	132.68	1.2	3.73	1.02
<=0	18099.6	5.7	<=0	<=0	21063.49	<=0	5.48	1.2	3995.78	1.96	<=0	<=0
<=0	<=0	<=0	<=0	<=0	21063.49	<=0	<=0	<=0	6.64	0.6	<=0	<=0
<=0	<=0	<=0	<=0	<=0	21063.49	<=0	<=0	<=0	<=0	<=0	<=0	<=0
<=0	325.21	3.21	<=0	<=0	21063.49	<=0	<=0	<=0	837.01	1.79	<=0	<=0

SEM	IL-6	SEM	IL-7	SEM	IL-8	SEM	IP-10	SEM	MCP-1	SEM	MIG	SEM
<=0	<=0	<=0	<=0	<=0	1070.24	<=0	<=0	<=0	267.29	1.67	<=0	<=0
<=0	0.91	0.22	<=0	<=0	1070.24	<=0	<=0	<=0	119.61	0.76	<=0	<=0
<=0	7427.79	2.56	<=0	<=0	1070.24	<=0	<=0	<=0	871.98	0.99	<=0	<=0
<=0	57.91	1.08	<=0	<=0	1070.24	<=0	<=0	<=0	684.18	0.69	<=0	<=0
<=0	455.13	1.34	<=0	<=0	1070.24	<=0	<=0	<=0	4529.06	1.57	<=0	<=0
<=0	1316.74	5.6	<=0	<=0	1070.24	<=0	<=0	<=0	6630.12	1.67	<=0	<=0
<=0	162.09	3.2	<=0	<=0	1070.24	<=0	<=0	<=0	65.26	0.42	<=0	<=0
<=0	<=0	<=0	<=0	<=0	1070.24	<=0	<=0	<=0	<=0	<=0	<=0	<=0
<=0	<=0	<=0	<=0	<=0	1070.24	<=0	<=0	<=0	<=0	<=0	<=0	<=0
<=0	<=0	<=0	<=0	<=0	1070.24	<=0	<=0	<=0	<=0	<=0	<=0	<=0
<=0	<=0	<=0	<=0	<=0	1070.24	<=0	<=0	<=0	<=0	<=0	<=0	<=0
<=0	0.62	0.32	<=0	<=0	1070.24	<=0	<=0	<=0	<=0	<=0	<=0	<=0

SEM	IL-6	SEM	IL-7	SEM	IL-8	SEM	IP-10	SEM	MCP-1	SEM	MIG	SEM
<=0	<=0	<=0	<=0	<=0	<=0	<=0	<=0	<=0	5.09	0.79	<=0	<=0
<=0	<=0	<=0	<=0	<=0	564.41	1.43	<=0	<=0	<=0	<=0	<=0	<=0
<=0	7.537	0.15	<=0	<=0	1070.24	<=0	<=0	<=0	436.8	28.01	<=0	<=0
<=0	1.427	0.1	<=0	<=0	1070.24	<=0	<=0	<=0	619.1	19.23	<=0	<=0
<=0	24.54	2.9	<=0	<=0	1070.24	<=0	<=0	<=0	826.4	19.09	<=0	<=0
<=0	1.474	0.14	<=0	<=0	1070.24	<=0	<=0	<=0	1012	13.77	<=0	<=0
<=0	3.659	0.55	<=0	<=0	<=0	<=0	<=0	<=0	360.1	12.59	<=0	<=0
<=0	2.437	0.29	<=0	<=0	686.21	2.3	<=0	<=0	218.6	16.07	<=0	<=0
<=0	0.6466	0.049	<=0	<=0	1070.24	<=0	<=0	<=0	333.2	25.65	<=0	<=0
<=0	0.2012	0.0015	<=0	<=0	619.31	1.23	<=0	<=0	41.66	1.66	<=0	<=0
<=0	5.675	0.515	<=0	<=0	1070.24	<=0	<=0	<=0	517.7	15.08	<=0	<=0
<=0	25.26	2.53	<=0	<=0	1070.24	<=0	<=0	<=0	349.5	15.17	<=0	<=0

MIP-1 alpha	SEM	MIP-1 beta	SEM	RANTES	SEM	TNF alpha	SEM	VEGF-A	SEM
27.08	0.1	38.04	1.98	<=0	<=0	2.8	0.44	2.38	1.87
11739.8	0.12	865.41	0.88	18.46	1.67	216.51	1.87	2.53	1.02
9.47	0.88	<=0	<=0	<=0	<=0	0.7	0.15	0.6	0.76
10.64	0.12	<=0	<=0	<=0	<=0	0.7	0.16	0.6	0.76
35.27	0.22	<=0	<=0	<=0	<=0	58.88	1.3	0.66	0.76

MIP-1 alpha	SEM	MIP-1 beta	SEM	RANTES	SEM	TNF alpha	SEM	VEGF-A	SEM
3.54	0.44	7.48	0.23	4.5	0.3	<=0	<=0	<=0	<=0
5.79	1.01	18.61	0.78	15.57	1.3	<=0	<=0	<=0	<=0
81707.42	1.76	1431.96	1.34	10.93	1	532.11	0.5	<=0	<=0
86.73	0.76	41.62	1.04	2.99	0.99	0.44	0.62	<=0	<=0
220.35	0.55	68.07	0.89	5.81	0.65	233.93	0.33	<=0	<=0
22111.55	1.91	1431.96	1.99	21.34	0.34	207.38	0.34	<=0	<=0
87.62	0.94	38.15	1.07	2.47	0.27	1.88	0.11	<=0	<=0
2.32	0.77	<=0	<=0	0.88	0.16	<=0	<=0	<=0	<=0
3.55	0.34	<=0	<=0	1.37	0.55	<=0	<=0	<=0	<=0
2.32	0.46	<=0	<=0	1.49	0.43	<=0	<=0	<=0	<=0
1.09	0.29	<=0	<=0	1.61	0.13	<=0	<=0	<=0	<=0
3.44	1.45	<=0	<=0	1.61	0.22	<=0	<=0	<=0	<=0

MIP-1 alpha	SEM	MIP-1 beta	SEM	RANTES	SEM	TNF alpha	SEM	VEGF-A	SEM
1.27	6	10	1.5	816.11	6	<=0	<=0	0.13	0.12
704.96	5.5	409	3.5	413.96	15.5	<=0	<=0	0.1	0.1
33.28	4.5	79.5	1.2	456.95	8.5	69.76	2.44	0.4	0.08
73.15	2.75	131.25	8.5	216.58	2.75	65	5	0.78	0.18
17.12	6.5	57	9	239.49	6.5	162.8	10.16	0.29	0.29
35.15	7.5	87.25	10	71.73	7.5	<=0	<=0	0.31	0.31
2.32	1.6	7.5	9	89.03	6	<=0	<=0	0.15	0.11
579.32	12	513.75	10.5	186.25	12	14.84	4.83	0.64	0.35
7.91	6.25	29.5	9	58.73	6.25	20.22	0.22	0.16	0.1
7.91	2.9	24.75	9.5	440.99	9	<=0	<=0	0.08	0.27
51.54	8	116.25	6.3	816.11	8	<=0	<=0	1	0.27
42.85	11.5	89.5	7.5	506.79	13.5	<=0	<=0	0.5	0.2
